# Supplementary material for: Efficacy and Safety of High-Dose Immunoglobulin-Based Regimen in Statin-Associated Autoimmune Myopathy: A Multi-Center and Multi-Disciplinary Retrospective Study
Source: J Clin Med. 2020 Oct 27;9(11):3454. doi: 10.3390/jcm9113454 (PMC7692610; doi:10.3390/jcm9113454)
Supplement: Supplementary file 1 [file jcm-09-03454-s001.pdf]

Table S1. Characteristics of the relapsed patients (*n*=3).

Pts.	Age at relapse	Months of follow-up at relapse	Clinical Features at relapse	CK level at relapse	Anti-HMGCR antibody titer at relapse	Ongoing therapy	Duration of drug-free period before relapse	Therapeutic changes
**P03**	75	18	Upper and lower limbs weakness (MRC 3/5), myalgia	2887 IU/L	420 UA/mL	None	9 months	Restarted monthly IVIG and MTX
**P05**	83	36	Upper and lower limbs weakness (MRC 3/5), myalgia	1485 IU/L	NA	None	12 months	Restarted steroid therapy (induction with 250 mg/day MP intravenously for 3 days). IVIG as maintenance therapy
**P13**	71	35	Upper and lower limbs weakness (MRC 2/5), myalgia	11344 IU/L	133 UA/mL	Monthly IVIG, MTX 10 mg/weekly	-	Started RTX monotherapy: RTX 1000 mg two weeks apart, then 1000 mg at third and sixth month, then 1000 mg every six months

Legend: CK, creatine kinase; anti-HMGCR, anti-hydroxy-methylglutaryl coenzyme A reductase; IV, intravenous; IVIG, immunoglobulins, MP, methylprednisolone; MTX, methotrexate; Pts, patients; RTX, rituximab; NA, not available.
